# *Coccidioides* Exposure and Coccidioidomycosis among Prison Employees, California, United States 

**DOI:** 10.3201/eid2106.141201

**Published:** 2015-06

**Authors:** Marie A. de Perio, R. Todd Niemeier, Gregory A. Burr

**Affiliations:** Centers for Disease Control and Prevention, Cincinnati, Ohio, USA

**Keywords:** Coccidioidomycosis, Coccidioides, Valley fever, occupational health, employees, prison, correctional facility, fungi, California, United States

## Abstract

Responding to a request by corrections agency management, we investigated coccidioidomycosis in prison employees in central California, a coccidioidomycosis-endemic area. We identified 103 cases of coccidioidomycosis that occurred over 4.5 years. As a result, we recommended training and other steps to reduce dust exposure among employees and thus potential exposure to *Coccidioides*.

Coccidioidomycosis, also known as Valley fever, is caused by inhalation of spores of the fungus *Coccidioides,* which grows in soil in semiarid areas. Coccidioidomycosis is endemic to the southwestern United States, the Central Valley of California, Mexico, and parts of Central and South America (*1*). An estimated 150,000 new infections occur annually in the United States (*2*). In disease-endemic areas, workers involved in soil disturbance, including agricultural, construction, and archeological workers, are at high risk for coccidioidomycosis (*1*).

As part of a health hazard evaluation requested by corrections agency management (*3*), we investigated the incidence of coccidioidomycosis among employees at 2 prisons in California’s Central Valley. To reduce exposure to *Coccidioides*, we recommended ways to improve coccidioidomycosis-related occupational health practices at the prisons.

## The Study

Prison A, a minimum–maximum security facility, and prison B, a minimum–medium security facility, employed ≈1,300 and 1,500 custody and support staff, respectively. The prisons are located on 640 acres in 2 (1 each) of 6 coccidioidomycosis-hyperendemic California counties. In those counties, annual case rates for coccidioidomycosis are consistently higher than rates for the state (*4*).

We identified confirmed cases of coccidioidomycosis among prison staff by using agency employee rosters and the Confidential Morbidity Reports from the California Department of Public Health for 2009–2013. We used the coccidioidomycosis case definition of the Council of State and Territorial Epidemiologists and defined a case as a clinically compatible illness that was laboratory confirmed (*5*) in a person employed at either prison at symptom onset. We calculated the crude (unadjusted) average annual incidence of coccidioidomycosis for prison A and B employees. Using information from the California Department of Public Health and from the corrections agency, we described personal and work characteristics of the employee case-patients.

During visits to the prisons in June 2013, we interviewed a convenience sample of 172 employees across all job categories about their work practices and exposures and met with staff to learn about dust mitigation efforts. As a public health response, according to Title 45 Code of Federal Regulations Part 46, this evaluation did not require review by an institutional review board.

**Figure 1:**
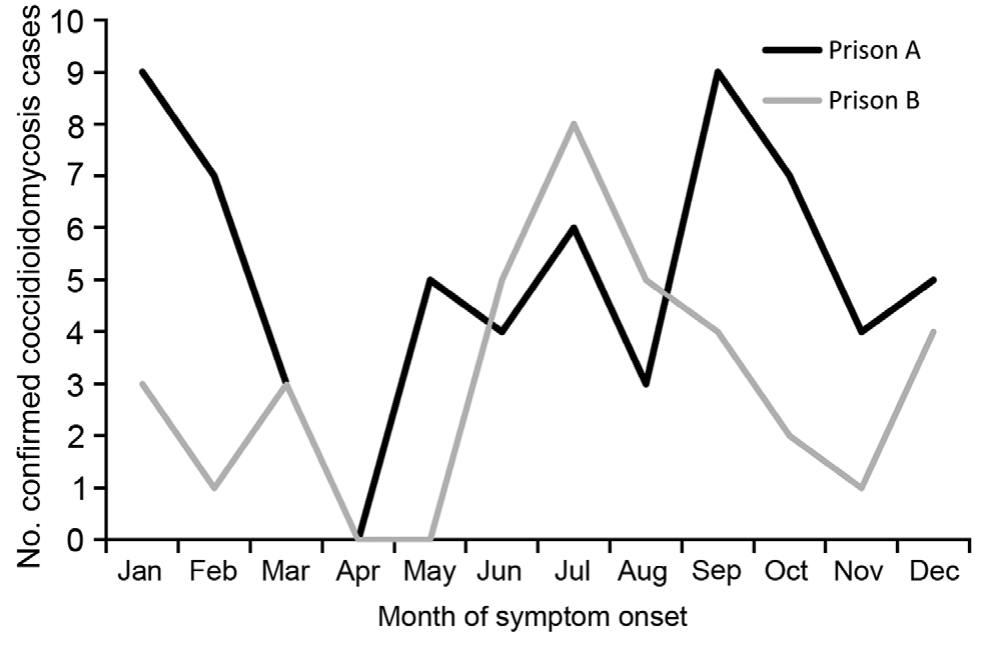
Number of coccidioidomycosis cases among prison A and B employees by month of symptom onset, California, USA, 2009–2012.

We identified 65 confirmed cases of coccidioidomycosis among prison A employees and 38 confirmed cases of among prison B employees from 2009 to mid-2013. These cases were reported by 9 counties where the employees resided. All but 3 employees resided in 1 of the 6 coccidioidomycosis-hyperendemic counties. For 2009–2012, the crude average annual incidence for prison A employees was 1,039 cases/100,000 employees, and for prison B employees, 511 cases/100,000 employees. The 12-month combined incidence for 2009–2012 showed no clear seasonal patterns (Figure 1).

Of 103 employees with confirmed illness, 84 (82%) were male; median age was 43 (range 24–67) years. The median time worked at the prison before symptom onset was 5 years (range 2 months–23 years). The most common job categories were custody (72%), health care (11%), administration (10%), and plant operations (2%).   We interviewed 172 (99%) of 173 invited employees at both prisons. Median age was 47 (range 25–74) years; 67% were male. Most (59%) reported their race as white, 5% as Filipino, and 3% as black or African American; 42% reported their ethnicity as Hispanic or Latino. The median number of years living in any coccidioidomycosis-hyperendemic California county was 35 (range 0.5–67) years. Thirty-five (20%) interviewed employees reported having >1 underlying medical condition that placed them at high risk for severe or disseminated disease, including asthma, emphysema, diabetes mellitus, immunosuppression resulting from medication, heart disease, kidney disease, and cancer requiring chemotherapy or radiation therapy.

Job categories of interviewed employees were custody (45%), administration (17%), plant operations (13%), health care (13%), education or vocational training (7%), and other (5%). Of interviewed employees, 171 (99%) reported outdoor work activities, including walking around prison grounds, performing security checks, patrolling, performing maintenance, and landscaping. The median time spent outdoors during the work day was 1.5 hours (range 10 minutes–9 hours). Forty-two (24%) employees reported conducting soil disruption activities during their job, including grid searches, digging for contraband, and drilling or digging for maintenance and repairs. The median time spent performing outside work was 2 hours per day (range 0–9 hours).

**Figure 2:**
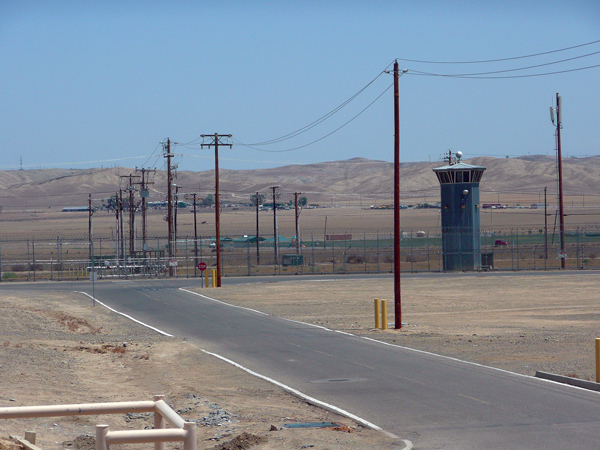
Prison B, located in an arid, coccidioidomycosis-hyperendemic area of the Central Valley of California, USA. Little natural vegetation grows on the grounds and in surrounding areas. Photograph courtesy of National Institute for Occupational Safety and Health.

During meetings with facilities and engineering staff at both prisons, we learned that efforts to reduce potential dust exposures on prison grounds already included wetting soil before soil disruption, reducing soil disking (shallow plowing), applying a soil stabilizer, and planting grass and other vegetation. Prison B’s grounds and surrounding areas have little natural vegetation (Figure 2).

## Conclusions

Our investigation revealed 103 cases of confirmed coccidioidomycosis in prison A and B employees from 2009 to mid-2013. The crude average annual incidence among prison employees seems to be higher than that reported among the general noninmate adult population in the surrounding counties (county A, 40 cases/100,000 persons; county B, 110 cases/100,000 persons) (J. Mohle-Boetani, pers. comm.; F. Tabnak, pers. comm.). However, comparisons of crude incidence rates between the prison employee and noninmate populations should be interpreted cautiously because of unmeasured confounding factors. We were unable to determine age-, sex-, and race-adjusted incidence rates for prison employees because these data were unavailable for this population; therefore, we could not make statistical comparisons. Also, reporting or testing bias is possible because of heightened awareness among employees and could have contributed to the difference in crude incidence rates.

Most employees with confirmed coccidioidomycosis were custody workers. However, because these workers represent most of the employee population, cases for this group did not seem to be disproportionate. We also could not determine if each confirmed coccidioidomycosis case in an employee was because of exposure at work or outside of work. Most prison employees with confirmed coccidioidomycosis resided in a coccidioidomycosis-hyperendemic area, and our interviews revealed that employees are probably exposed to *Coccidioides* at work and outside of work. Almost all interviewed employees at each prison reported spending time outdoors at work, and almost one third, specifically custody and plant operations employees, reported work involving soil disruption.

The prisons face ongoing challenges of maintaining or restoring vegetation and grass on the grounds because of water restrictions. Environmental mitigation efforts, such as reducing soil disking, paving roads, and wetting soil before disturbing it, can reduce dust levels and therefore may lower the risk for localized airborne dispersion of *Coccidioides* spores. However, little data exist to demonstrate the effectiveness of these measures in reducing airborne dispersal and occupational coccidioidomycosis (*6*). Airborne spores can travel for miles, and focal *Coccidioides* sites may be small and unevenly distributed within coccidioidomycosis-endemic areas (*6**–**8*). Environmental mitigation measures neither eradicate the organism from soil nor prevent exposure to dust from areas outside the prison grounds. Nevertheless, reducing dust is a reasonable risk-reduction strategy for addressing occupational coccidioidomycosis.

On the basis of our findings, we recommended that prison management weigh the advantages and disadvantages of various environmental mitigation efforts to reduce dust exposures. Our recommendations included providing employees with education and training about coccidioidomycosis symptoms and transmission, risk factors for disseminated disease, and ways to minimize exposures; closing the prison yards during dust storms; and using respirators approved by the National Institute for Occupational Safety and Health as part of a respiratory protection program for employees who must work outside during unusually dusty days or who may disturb soil. The recently available coccidioidal spherulin skin test may also be a potentially useful tool for identifying at-risk employees.
